# Water-Induced Breaking of Interfacial Cohesiveness in a Poly(lactic acid)/Miscanthus Fibers Biocomposite

**DOI:** 10.3390/polym13142285

**Published:** 2021-07-12

**Authors:** Nicolas Delpouve, Hajar Faraj, Clément Demarest, Eric Dontzoff, Marie-Rose Garda, Laurent Delbreilh, Benjamin Berton, Eric Dargent

**Affiliations:** University of Rouen Normandie, INSA Rouen, CNRS, GPM, 76000 Rouen, France; hajar.faraj@agroparistech.fr (H.F.); clement.demarest@univ-rouen.fr (C.D.); eric.dontzoff@univ-rouen.fr (E.D.); marie-rose.garda@univ-rouen.fr (M.-R.G.); laurent.delbreilh@univ-rouen.fr (L.D.); benjamin.berton@univ-rouen.fr (B.B.); eric.dargent@univ-rouen.fr (E.D.)

**Keywords:** PLA, mechanical properties, thermal stability, calorimetry, degradation

## Abstract

The impact of the immersion in water on the morphology and the thermomechanical properties of a biocomposite made of a matrix of poly (lactic acid) (PLA) modified with an ethylene acrylate toughening agent, and reinforced with miscanthus fibers, has been investigated. Whereas no evidence of hydrolytic degradation has been found, the mechanical properties of the biocomposite have been weakened by the immersion. Scanning electron microscopy (SEM) pictures reveal that the water-induced degradation is mainly driven by the cracking of the fiber/matrix interface, suggesting that the cohesiveness is a preponderant factor to consider for the control of the biocomposite decomposition in aqueous environments. Interestingly, it is observed that the loss of mechanical properties is aggravated when the stereoregularity of PLA is the highest, and when increasing the degree of crystallinity. To investigate the influence of the annealing on the matrix behavior, crystallization at various temperatures has been performed on tensile bars of PLA made by additive manufacturing with an incomplete filling to enhance the contact area between water and polymer. While a clear fragilization occurs in the material crystallized at high temperature, PLA crystallized at low temperature better maintains its properties and even shows high elongation at break likely due to the low size of the spherulites in these annealing conditions. These results show that the tailoring of the mesoscale organization in biopolymers and biocomposites can help control their property evolution and possibly their degradation in water.

## 1. Introduction

The current environmental situation is negatively marked by air pollution and waste multiplication. In this context, material scientists look for solutions that keep the functionalities at use and have a minimum environmental impact [1]. Biocomposites properties have thus been investigated with a large variety of matrices and vegetal fiber reinforcements [2,3]. Poly lactic acid (PLA) notably is one of the main actors in biomaterials industrialization, targeting the packaging market. It is characterized among biopolymers by its ease of processing and its rigidity. On the other hand, its elongation at break is low and its impact strength is quite poor [4]. As possible strategies to minimize these drawbacks, toughening agents have been added post-polymerization to the PLA matrix, thermoplastic elastomers, glycidyl esters, and polyolefins among others [5,6].

Water-induced degradation is a major phenomenon to study since a significant amount of wastes that escape from the recycling or incineration chains are rejected into oceans or soils [7]. In both cases, the future of the material is strongly dependent on the aptitude of the material to fragment and degrade under the water action. Most of the studies previously reported evidence of water-induced degradation at the molecular level [8,9,10,11], with careful consideration of the hydrolytic mechanisms, that is, the chain scission or the appearance of new functionalities. Those were shown by means of pH-metric measurements, gel permeation chromatography, and infrared spectroscopy [8,9,10,11].

Furthermore, the role of the mesoscale organization is not to be neglected since most biopolymer matrices are semi-crystalline. The morphology, as well as the microstructure, can significantly impact the residual macroscopic properties. For example, the diffusion of water in PLA is known to be influenced by both the space-filling of spherulites and the content of rigid amorphous fraction (RAF) [12]. Furthermore, the fiber/matrix interface of the biocomposite also needs to be optimized to ensure decent properties. Finally, the first steps of the degradation process will be governed by the fragmentation mechanisms, which are influenced by the mesoscale organization.

In this work, a PLA matrix has been reinforced with fibers of miscanthus (FM) that exhibit similar characteristics to bamboo fibers while being more accessible, since it is locally cultivated. An ethylene acrylate (EA) bio-approved toughening agent has been added to the mixture to partly solve the PLA mechanical deficiencies, as the ethylene part plays a shock-absorbent role, and to slightly improve the adhesion at the fiber/matrix interface. The resulting PLA_EA_FM material has been characterized by means of thermal analysis, mechanical tests, and electron microscopy. The material structure and macroscopic properties have been investigated before and after immersion in water, for amorphous or semi-crystalline PLA_EA_FM matrices, and for PLAs with different stereoregularity.

To understand the role of annealing conditions on the future degradation of the PLA_EA_FM biocomposite, we have previously applied those conditions on amorphous PLA. It is expected that the important number of hydrophilic functionalities in the lignin, cellulose, and hemicelluloses parts of the FM create preferential channels that facilitate sorption and diffusion of water. Therefore, additional neat PLA tensile bars have been processed by additive manufacturing and annealed at various temperatures with the aim of tailoring the spherulite size and the RAF content. The filling of the manufactured bars was intentionally partial to facilitate the water diffusion and speed up the degradation. The influence of the annealing conditions on the post-immersion properties is implemented in the discussion regarding the biocomposite behavior.

## 2. Materials and Methods

### 2.1. Materials

Two PLA matrices were used in this study for the manufacturing of the bio-composite. PLA 4043D^®^ was industrial-grade and purchased from NatureWorks^®^ (Minnetonka, MN, USA) with a 98% l-enantiomer [13]. Purasorb PL 24^®^ from Purac^®^ is a grade that contains 99.2% l-enantiomer, therefore, it crystallizes faster. The miscanthus fibers were purchased by AMP^®^ (WuZhen, China). Before processing, the fibers were washed then sieved using a Retsch^®^ instrument (Haan, Germany) and finally dried in a furnace at 55 °C. The structural integrity of the fibers is maintained for a processing temperature less than 200 °C. In all bio-composites, the fiber weight percentage is 20%. The chosen fiber ratio allows one to obtain mechanical reinforcement properties and increasing the contact area between the matrix and the fiber. The additive is a bio-approved ethylene copolymer Biomax Strong 120^®^ purchased by DuPont^®^ (Wilmington, DE, USA). A 5% copolymer was incorporated into the total mass of the bio-composite.

### 2.2. Additive Manufacturing of PLA

PLA4043D^®^ tensile bars were made in a Lulzbot Taz^®^ machine (Fargo, ND, USA). The shape and mesh designs are given in Supporting Information (Figure SI(1a)). The bar had no shell and was filled at only 90% to allow easier water diffusion. It was only supported by an external wall of 0.5 mm thickness. The temperature of the fused deposit was 205 °C and the temperature of the plate was 60 °C. The speed of the deposit was 40 mm s^−1^ and its width was 0.5 mm. A total of 20 layers were printed, each layer being 0.2 mm thick, for a total thickness of 4 mm. The solicited domain was therefore 70 × 10 × 4 mm^3^.

### 2.3. Processing of the PLA-Based Biocomposite

The blending was initiated in a Hobart^®^ laboratory mixer, then the materials were dried in a furnace at 55 °C for at least three hours. The compoundage was finalized in an IQAP-LAP E/30-25 D^®^ single screw extruder. The extrusion was performed at 35 rpm with the following *T*_min_/*T*_max_ (°C) temperature profile: 160/175; 170/180; 180/190; 190/200. The obtained plastic rods were crushed in a CMB ML 300 F STD^®^ grinder, and then injection molded (195 °C and 1300 bars pressure) in a BATTENFELD BA 350 CD^®^ press to obtain mechanical tests bars according to the NF EN ISO 294-3 norm. The pictures of the biocomposite after each step of the processing are provided in Supporting Information (Figure SI(1b)).

### 2.4. Crystallization and Immersion

All the materials were dried at 50 °C for 30 h. The biocomposite was put in a furnace under air for 48 h at 100 °C. On the other hand, the tensile bars obtained by additive manufacturing were crystallized at 80 and 135 °C for 7 days to highlight how the thermal treatment influences the macroscopic properties depending on the induced crystalline morphology. Indeed, these two crystallization temperatures were chosen to favor either germination or crystal growth, respectively. The immersion in water was done at ambient temperature with regular refilling for various times ranging from 24 h to 2 years. The follow-up of the water-induced degradation of the biocomposite was carried out using gravimetric (Mettler Toledo^®^, Viroflay, France) balance and pH-metric measurements.

### 2.5. Material Characterization

Thermogravimetric analyses (TGA) were performed at 10 K/min from 30 to 700 °C in a Thermal Analysis ^®^ Discovery, Guyancourt, France, apparatus under a nitrogen flow of 25 mL/min at a scanning rate of 10 K min^−1^. The calibration in temperature was done using the Curie point of nickel as a reference.

Modulated temperature differential scanning calorimetry (MT-DSC) analyses were performed in heat-only conditions at 2 K min^−1^ with an amplitude of +/− 0. 318 K and a period of 60 s in a Thermal Analysis^®^ Q2000 Guyancourt, France, apparatus. The calibration in temperature was carried out using indium standard when the calibration in heat capacity was performed from the analysis of a sapphire sample. Before any analysis, the material was first heated above its glass transition to suppress physical aging effects.

Test bars (70 × 10 × 4 mm^3^) were solicited in tensile mode at room temperature at 0.1 mm min^−1^ in an INSTRON^®^ S3365 device (Norwood, MA, USA) equipped with a 1 kN force sensor. The same apparatus and sensors were used for 3-point flexural tests at 1 mm min^−1^, with the distance between each support equal to 50 mm.

The material toughness was investigated by Charpy shock tests in a CEAST^®^ apparatus with a 15 Joules hammer, the pendulum speed defined by the purchaser being 2.9 m s^−1^. The samples were 10 mm large and 3 mm thick, the V notch drawn at the opposite of the hit was 1.75 mm depth.

Scanning electron microscopy pictures of the biocomposite were acquired using the Zeiss^®^ Leo 1530 Marlyle Roi, France, Gemini column apparatus. The InLens and SE2 sensors were both used for picture acquisition. The electrons were accelerated at 10 and 20 keV, and the work distance varied between 11 and 16 mm. The sample was fractured in liquid nitrogen, then polished and fixed on the support with a conducting silver lacquer glue. Finally, the sample was coated with 10 nm gold to prevent irradiation effects.

## 3. Results and Discussion

### 3.1. Post-Immersion Properties of the Additive Manufactured PLA

**Figure 1:**
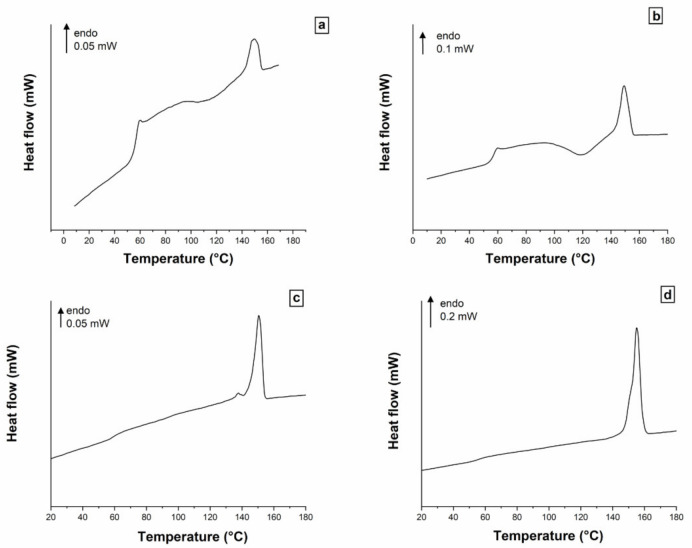
MT-DSC responses of (**a**) amorphous PLA before immersion, (**b**) amorphous PLA after 2 months of immersion FESEM, (**c**) PLA crystallized 7 days at 80 °C and immersed 2 months, (**d**) PLA crystallized 7 days at 135 °C and immersed 2 months.

The behavior of additive manufactured neat PLA was investigated before and after immersion. Figure 1 presents the MT-DSC responses of amorphous and semi-crystalline PLA that were immersed for 2 months. By comparing first the two amorphous PLAs (Figure 1a,b), one can observe that both signals exhibit the same characteristic events, the endothermic step associated with the glass transition, the exothermic peak of cold-crystallization, and finally the endothermic peak of melting. In both cases, the enthalpies of cold-crystallization and melting are equal, proving that PLA stays amorphous after immersion. However, the enthalpy of cold-crystallization is higher when the PLA is immersed. This shows that the nucleation has been enhanced. Regarding semi-crystalline PLAs (Figure 1c,d), the absence of cold-crystallization peak reveals that both samples reached their maximum degree of crystallinity *X*_c_. As expected, the amplitude of the glass transition decreases in both cases in comparison with amorphous PLAs since the content of the amorphous phase is lower. PLA crystallized at 135 °C exhibits only a melting peak, which is attributed in literature to the melting of α crystals formed at high crystallization temperature [14]. On the other hand, the behavior of the PLA crystallized at 80 °C is not what is usually reported. Most often, one can see a low-intensity exothermic peak that precedes the melting peak. This is the signature of the reorganization of imperfect α’ crystals formed at low crystallization temperature into α crystals, which subsequently melt [15]. In the present study, a double melting peak is recorded. Such calorimetric response has previously been observed in neat PLA annealed in a furnace [12]. It is assumed that the crystallization process is heterogeneous and that thermal gradients exist through the material thickness. Thus, the double peak is interpreted as the signature of the coexistence of α’ and α crystals.

Nevertheless, the crystallization procedures do not induce the same microstructure. As showed in Table 1, and previously reported in the literature, crystallization at 80 °C leads to a higher content of rigid amorphous fraction *X*_RAF_ whereas the crystallization at 135 °C favors the decoupling between both crystalline and amorphous phases [16,17], thus leading to a high content of mobile amorphous fraction *X*_MAF_.

**Figure 2:**
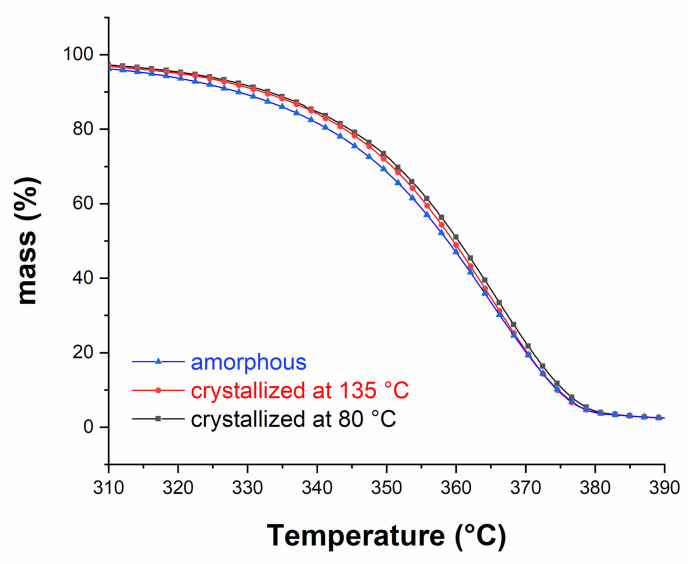
TGA curves of amorphous and semi-crystalline PLAs before immersion.

According to the TGA measurements performed before immersion, and shown in Figure 2, both crystallization procedures slightly improve the thermal stability of the bulk PLA (the characteristic temperatures *T*_w%_ and *T*_max_ corresponding to representative mass-loss percentage w% and to the maximum degradation rate, respectively, are given in Table 1). The effects are not spectacular though. In all cases, the degradation occurs in one step and the residual mass is about 1–2%. The crystallization shifts *T*_5%_ 5 °C towards higher temperatures, while the other characteristic temperatures are quite similar.

In contrast with TGA results, the mechanical tests reveal that the behavior of semi-crystalline PLA is dependent on the annealing conditions. In comparison with amorphous PLA, the PLA crystallized at 135 °C essentially lost its mechanical properties (Table 2). Although its Young’s modulus is barely affected, the stress at break and the strain at break both decrease from 30 to 6.5 MPa, and from 4 to 1%, respectively. Therefore, the crystallization at 135 °C aggravated the brittle character of PLA. On the other hand, the PLA crystallized at 80 °C exhibits a Young’s modulus of about 800 MPa, a stress at break of 20 MPa, and a strain at break of 3.5%. This ability to undergo the deformation has been reported in the literature for small-sized crystals, like those generated at 80 °C, and explained by their aptitude to transfer the stress more successfully than big crystalline lamellae [18]. It is likely that the specific design of the tensile bars, which includes many holes, and thus localized stress concentration areas, contributes to magnifying the impact of the crystallization protocol. The imperfect decoupling between crystal and amorphous PLA might also contribute to better stress transfer but it has to be proven. Furthermore, the Charpy results (Table 2) lead to the same conclusions regarding the influence of the crystallization procedure. The average toughness is almost identical for the amorphous PLA and the PLA crystallized at 80 °C, about 16 kJ m^−2^, but it falls to 4 kJ m^−2^ for the PLA crystallized at 135 °C. It has to be mentioned that the immersion of PLA into water has almost no effect at all on its mechanical properties. After two years of immersion, the variations are negligible in terms of Young’s modulus, stress at break, and strain at break for all PLAs. It seems that PLA is almost insensitive to the action of water at ambient temperature. These results are consistent with those obtained by Moetazedian et al. [10], who report that the signatures of degradation are barely discernible for a temperature of immersion lower than 37 °C.

### 3.2. Post-Immersion Properties of the Biocomposites

The analyzed biocomposites are based on PLA4043D and PLLA. Two of them did not undergo thermal treatment after the process. A third one, whose matrix is PLLA, was annealed in a furnace under air for 48 h at 100 °C (see paragraph 2.4). These three materials are named PLA4043D_EA_FM, PLLA_EA_FM, and annealed PLLA_EA_FM in the subsequent sections. Post-processing MT-DSC tests were carried out to determine their degree of crystallinity *X*_c_ (Table 3). PLA4043D_EA_FM is almost amorphous (*X*_c_ = 5%) whereas *X*_c_ = 35% in PLLA_EA_FM shows that it can crystallize during the process, even without further annealing, due to its higher stereoregularity. Nevertheless, the crystallization is boosted by the annealing. Thus, *X*_c_ = 60% in annealed PLLA_EA_FM.

**Figure 3:**
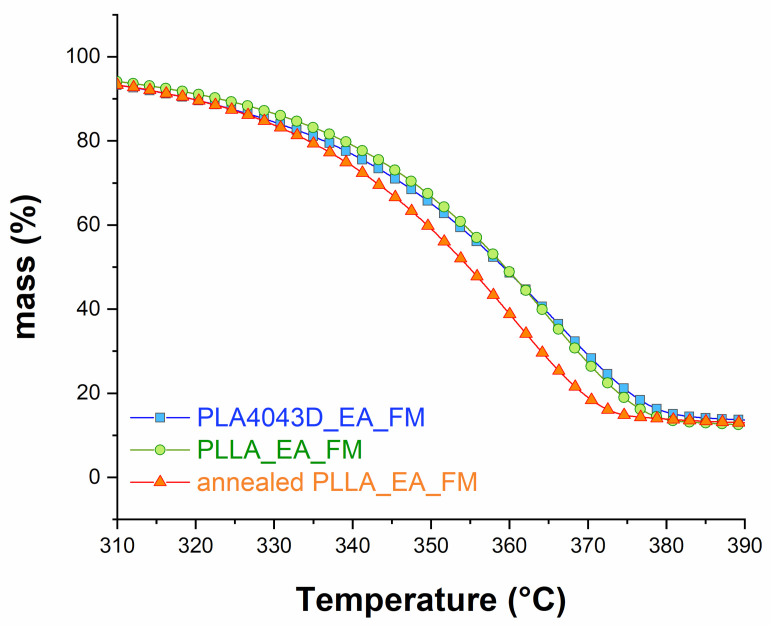
TGA curves of the biocomposites before immersion.

TGA analyses on the biocomposites before immersion are presented in Figure 3. The degradation occurs in one step for the three biocomposites. Interestingly, the annealed PLLA_EA_FM exhibits weaker resistance against thermal degradation. In particular, *T*_50%_ and *T*_max_ are recorded to be 5 °C lower in comparison with the other biocomposites (see Table 3). These results come surprisingly in opposition with the improvement of thermal stability under annealing that has been reported for the additive manufactured PLA. Apparently, the annealing of the PLLA_EA_FM biocomposite is slightly detrimental to its structural integrity.

**Figure 4:**
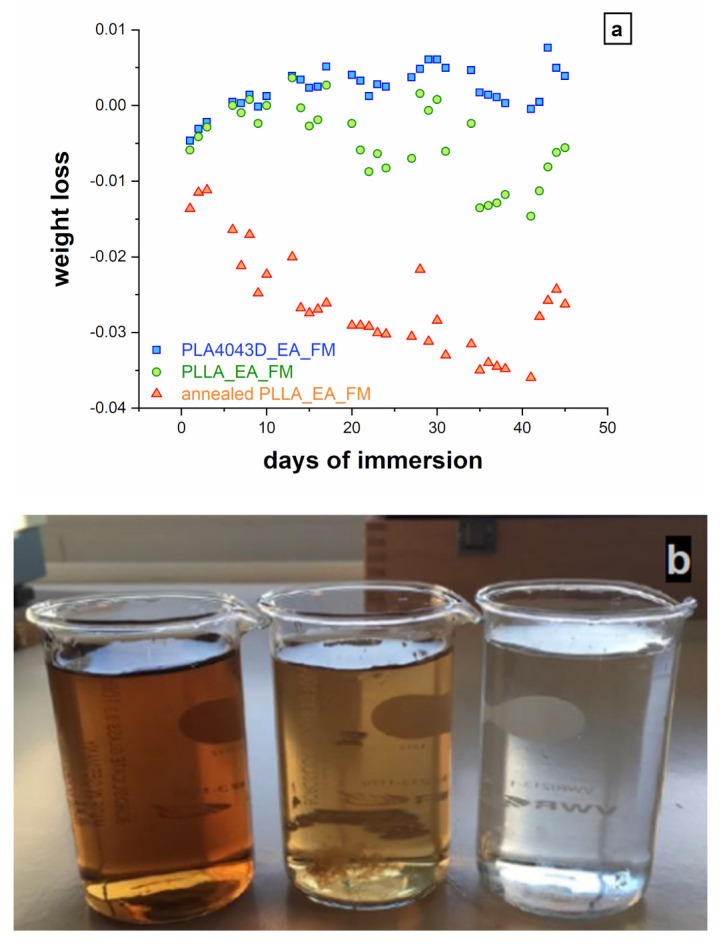
(**a**) evolution of the composite mass with the time of immersion, (**b**) solutions taken from the immersion medium. From left to right: annealed PLLA_EA_FM, PLLA_EA_FM, PLA4043D_EA_FM.

The results of gravimetric measurements along with the immersion are presented in Figure 4a. After 40 days in water, the mass of PLA4043D_EA_FM did not evolve, showing the stability of the composite in aqueous conditions. On the other hand, the annealed PLLA_EA_FM lost 4% of its initial mass, which evidences a lack of resistance to the action of water. The allure of the solutions after 7 months of immersion (Figure 4b) is consistent with these variations. While the solution was still transparent in the case of PLA4043D_EA_FM, it became yellowish, and even brownish for PLLA_EA_FM, and annealed PLLA_EA_FM respectively, suggesting that some fiber constituents have migrated into the water, lignin in particular. It is worth mentioning that no variation of the pH was recorded in the immersion solution. The hydrolytic degradation of polyester is expected to generate carboxylic acid functions, thus induce the acidification of the solution [8]. Our results suggest, on the other hand, that the composite has not been (or only minimally) hydrolyzed. Furthermore, the PLA matrix previously analyzed has not shown particular sensitivity to the water. More likely is the mass loss related to the desegregation of the composite at the fiber/matrix boundaries.

**Figure 5:**
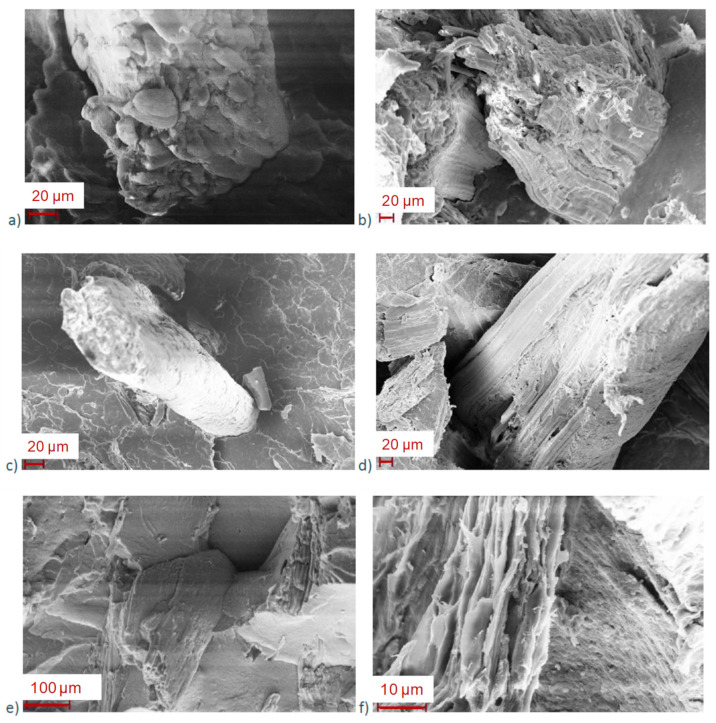
SEM pictures of (**a**) PLA4043D_EA_FM before immersion, (**b**) PLA4043D_EA_FM after 7 months of immersion, (**c**) PLLA_EA_FM before immersion, (**d**) PLLA_EA_FM after 7 months of immersion, (**e**) annealed PLLA_EA_FM before immersion, (**f**) annealed PLLA_EA_FM after 7 months of immersion.

SEM pictures were taken before (Figure 5a,c,e) and after (Figure 5b,d,f) immersion. Regarding non-immersed samples, the surface of PLA4043D_EA_FM is regular, with well-dispersed fibers and good adhesion between fibers and matrix. For PLLA_EA_FM, the surface is less regular, and some fiber agglomerates are detected. When PLLA is annealed, the fiber dispersion is even worse in the composite, and some fissures and microcracks appear. In contrast with the PLA composite, there is no clear adhesion between the fiber and the matrix. Instead, the matrix creates a block shape structure close to the fiber. After 7 months of immersion, in the PLA4043D_EA_FM composite, the fiber kept its structure and its good adhesion to the matrix, whereas some holes can be distinguished at the interface of PLLA_EA_FM, showing a loss of cohesiveness. For the annealed PLLA_EA_FM, one can immediately observe the irregularity of the surface and the cracking of the matrix. This morphology is favorable to water diffusion. Moreover, the cell wall of the fiber was clearly damaged in consistence with the gravimetric measurements. This reveals that the degradation has advanced enough to expect consequences on the mechanical properties.

**Figure 6:**
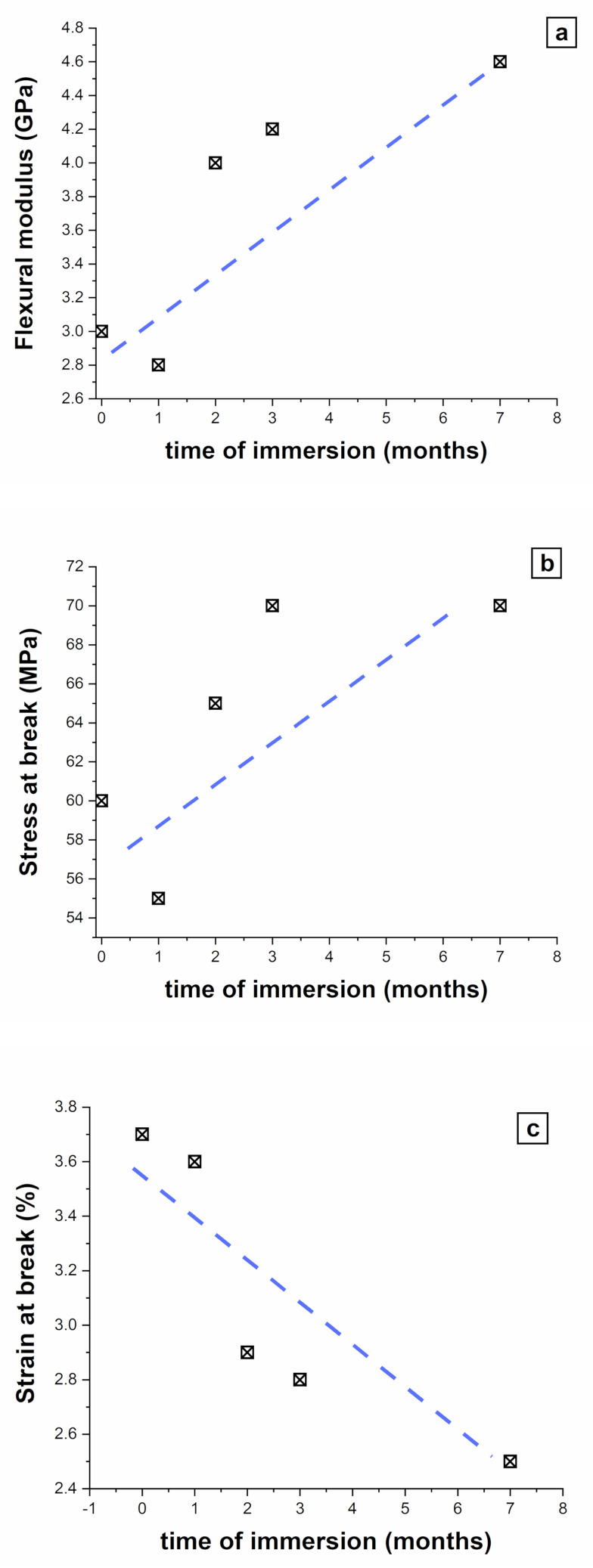

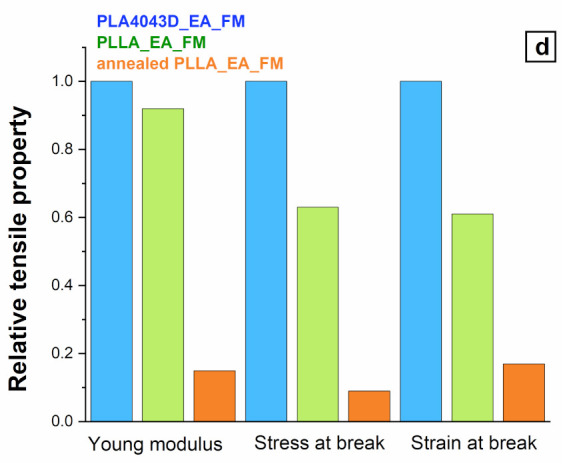
Flexural modulus (**a**), stress at break (**b**), and strain at break (**c**) from flexural tests, as a function of the immersion time. (**d**) Relative tensile properties of the 7-month immersed materials calculated by the ratio between any mechanical property of the biocomposite and the same mechanical property of PLA4043D_EA_FM.

Figure 6 presents the results from mechanical tests, both in flexion (Figure 6a–c) and tension (Figure 6d). One can see that the immersion in water induces an increase in the flexural modulus (Figure 6a), an increase of the stress at break (Figure 6b), and a decrease in the elongation at break (Figure 6c) in PLA4043D_EA_FM. These results indicate altogether that the material rigidifies and fragilizes with time. It is worth mentioning that the results from Charpy tests (not shown here) led to heterogeneous results, which did not allow us to conclude on the toughness evolution with time although it seems to slightly increase. In Figure 6d, the tensile properties of the 7-month immersed biocomposites are compared. Obviously, the Young’s modulus, the stress at break, and the strain at break are the highest in the PLA4043D_EA_FM and the lowest in the annealed PLLA_EA_FM, revealing severe degradations of the mechanical properties for the latter. These results are consistent with those obtained from gravimetric, TGA, and SEM measurements, which show that the annealing is detrimental to the cohesiveness of the fiber/matrix interface, leading to weakened thermal stability, and faster weight loss in water. It could seem counterintuitive that the degradation occurs faster in the biocomposite with the highest degree of crystallinity, but it can be explained by the embrittlement of the matrix. Indeed, with the growth of the crystalline phase, the fragile character of PLLA becomes even more pronounced. As a consequence, microcracks can emerge in the matrix. This facilitates the water diffusion, thus the decohesion of the fiber/matrix interface. Finally, the insulated fiber is progressively damaged by water, which leads to a global loss of mechanical properties. These results show that, by playing on the matrix structure, in particular the organization at the mesoscale, one can shift the properties of a biocomposite, towards stability over time, or, in contrast, towards easier degradation.

## 4. Conclusions

The volume accessible for the water diffusion is showed to be a major factor in the process of degradation in aqueous environments. We observe that it can be influenced by playing on the matrix stereoregularity, the crystalline morphology, and the fiber/matrix interface of biocomposites. Besides, any structural modification that damages the organization at the mesoscale accelerates the loss of property and probably benefits the bio-assimilation through faster degradation. This explains why the higher sensitivity to the water immersion has been surprisingly detected from mechanical tests in semi-crystalline biocomposites. Although the crystalline phase is denser than the amorphous phase, the generation of thick crystalline lamellae at high annealing temperature aggravates the PLA brittleness. Therefore, in the biocomposites, more cracks develop at the fiber/matrix interface, as shown by scanning electron microscopy. On the other hand, the crystallization conditions that favor the space-filling of the volume by spherulites of small size also strengthen PLA, which may be considered for better resistance against the action of water.

## Figures and Tables

**Table 1:** TGA characteristic degradation temperatures and MT-DSC fraction contents.

	TGA (°C)				MT-DSC		
	*T*_5%_	*T*_10%_	*T*_50%_	*T*_max_	*X*_c_	*X*_MAF_	*X*_RAF_
Amorphous PLA	316	329	358	366	0	1	0
PLA crystallized at 80 °C	321	333	360	367	0.33	0.31	0.36
PLA crystallized at 135 °C	320	331	359	367	0.50	0.35	0.15

**Table 2:** Young’s Modulus (E), stress at break (σ), strain at break (ε), and toughness (*K*_lc_) from mechanical tests.

	Tensile Tests			Charpy
	E (MPa)	σ Break (MPa)	ε Break (%)	*K_lc_* (kJ m^−2^)
Amorphous PLA	920	30	4.0	16
Amorphous PLA (2 years of immersion)	850	27	4.0	/
PLA crystallized at 80 °C	780	20	3.5	16
PLA crystallized at 80 °C (2 years of immersion)	880	18	4.0	/
PLA crystallized at 135 °C	950	6.5	1.0	4
PLA crystallized at 135 °C (2 years of immersion)	1000	6.0	1.0	/

**Table 3:** Thermal properties of the biocomposites.

	TGA (°C)				MT-DSC
	*T*_5%_	*T*_10%_	*T*_50%_	*T*_max_	*X*_c_
PLA4043D_EA_FM	302	319	359	365	0.05
PLLA_EA_FM	305	323	359	365	0.35
annealed PLLA_EA_FM	302	319	354	360	0.60

## Data Availability

Not applicable.

## Supplementary Materials

The following are available online at https://www.mdpi.com/article/10.3390/polym13142285/s1, Figure S1: (a) Scheme of the mesh design for the additive manufacturing of PLA tensile bars. (b) Pictures from the different step of the biocomposite processing including the fiber incorporation, the extrusion, the grinding and finally the injection.
