# Supplementary material for: Water-Induced Breaking of Interfacial Cohesiveness in a Poly(lactic acid)/Miscanthus Fibers Biocomposite
Source: Polymers (Basel). 2021 Jul 12;13(14):2285. doi: 10.3390/polym13142285 (PMC8309251; doi:10.3390/polym13142285)
Supplement: Supplementary file 1 [file polymers-13-02285-s001.zip › polymers-1300501-supplementary.pdf]

a)

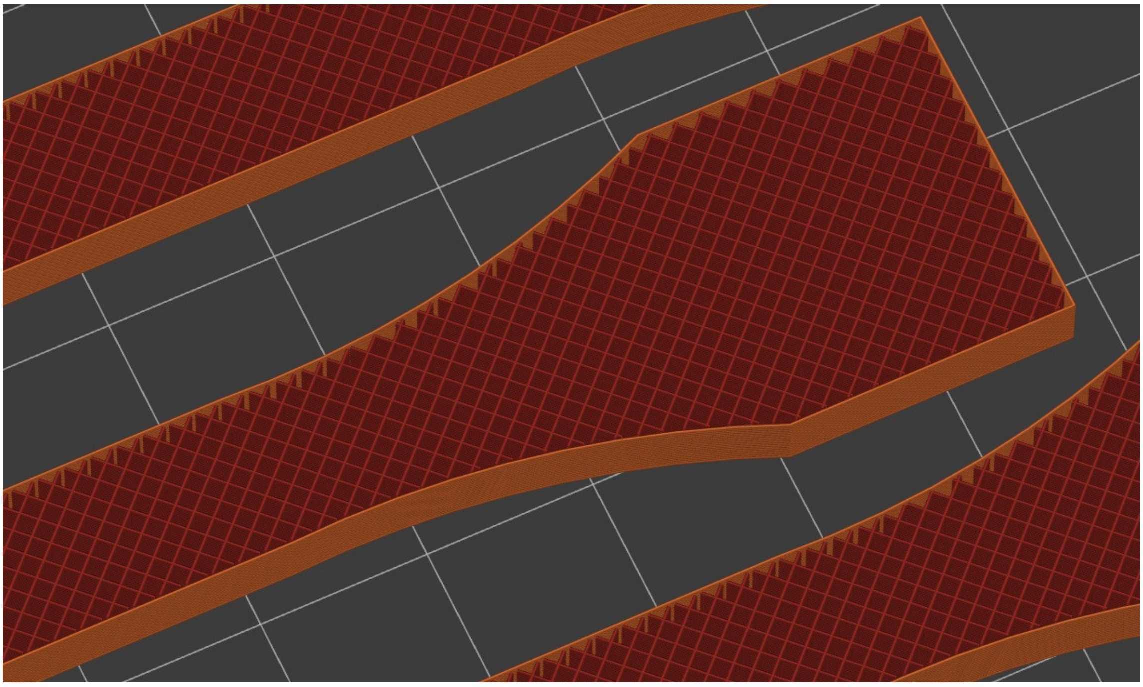

b)

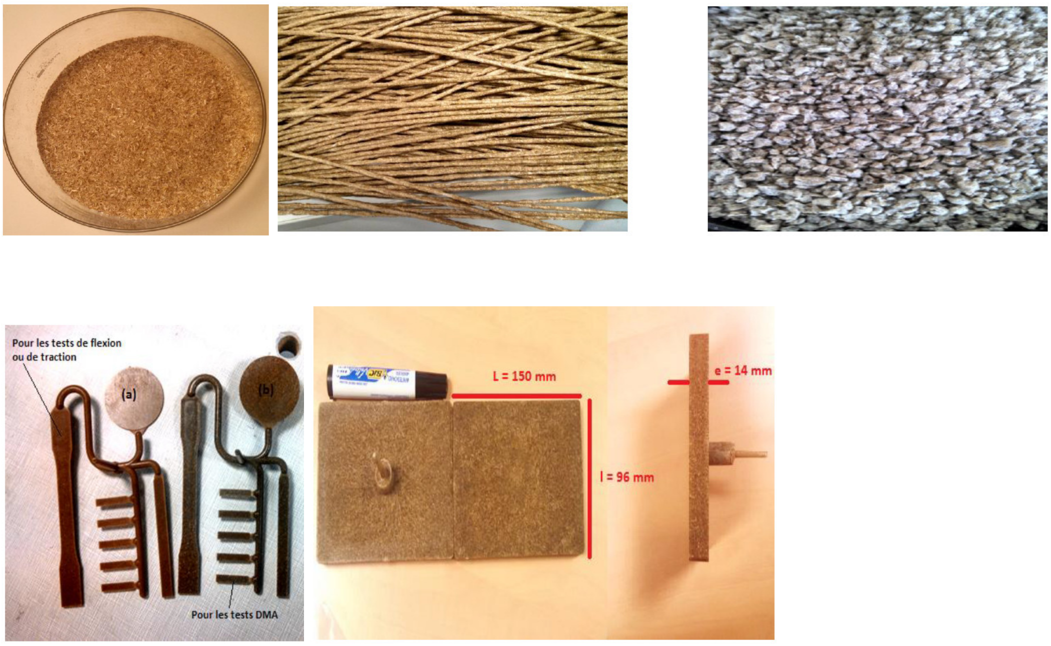

Figure SI.1. (a) Scheme of the mesh design for the additive manufacturing of PLA tensile bars. **(b)** Pictures from the different step of the biocomposite processing including the fiber incorporation, the extrusion, the grinding and finally the injection.
